# Diabetes is associated with familial idiopathic normal pressure hydrocephalus: a case–control comparison with family members

**DOI:** 10.1186/s12987-020-00217-0

**Published:** 2020-09-15

**Authors:** Joel Räsänen, Joel Huovinen, Ville E. Korhonen, Antti Junkkari, Sami Kastinen, Simo Komulainen, Minna Oinas, Cecilia Avellan, Janek Frantzen, Jaakko Rinne, Antti Ronkainen, Mikko Kauppinen, Kimmo Lönnrot, Markus Perola, Anne M. Koivisto, Anne M. Remes, Hilkka Soininen, Mikko Hiltunen, Seppo Helisalmi, Mitja I. Kurki, Juha E. Jääskeläinen, Ville Leinonen

**Affiliations:** 1 Department of Neurosurgery, Kuopio University Hospital, P.O.Box 100, 70029 Kuopio, KYS Finland; 2 Institute of Clinical Medicine-Neurosurgery, University of Eastern Finland, Kuopio, Finland; 3 Department of Neurosurgery, University of Helsinki, Helsinki, Finland; 4 Department of Neurosurgery, Helsinki University Hospital, Helsinki, Finland; 5 Clinical Neurosciences, Department of Neurosurgery, University of Turku, Turku, Finland; 6 Department of Neurosurgery, Turku University Hospital, Turku, Finland; 7 Department of Neurosurgery, Tampere University Hospital, Tampere, Finland; 8 Unit of Clinical Neuroscience, Neurosurgery, University of Oulu and Medical Research Center, Oulu University Hospital, Oulu, Finland; 9 National Institute for Health and Welfare, Helsinki, Finland; 10 University of Helsinki, Helsinki, Finland; 11 Institute of Clinical Medicine–Neurology, University of Eastern Finland, Kuopio, Finland; 12 Unit of Clinical Neuroscience, Neurology, University of Oulu, Oulu, Finland; 13 Medical Research Center, Oulu University Hospital, Oulu, Finland; 14 Institute of Biomedicine, University of Eastern Finland, Kuopio, Finland; 15 Analytical and Translational Genetics Unit, Department of Medicine, Massachusetts General Hospital, Boston, USA; 16 Program in Medical and Population Genetics, Broad Institute of MIT and Harvard, Cambridge, USA; 17 Stanley Center for Psychiatric Research, Broad Institute for Harvard and MIT, Cambridge, USA

**Keywords:** Normal pressure hydrocephalus, Familial, Comorbidities, Diabetes, Depression, Genetics, *SFMBT1*

## Abstract

**Background:**

The pathophysiological basis of idiopathic normal pressure hydrocephalus (iNPH) is still unclear. Previous studies have shown a familial aggregation and a potential heritability when it comes to iNPH. Our aim was to conduct a novel case-controlled comparison between familial iNPH (fNPH) patients and their elderly relatives, involving multiple different families.

**Methods:**

Questionnaires and phone interviews were used for collecting the data and categorising the iNPH patients into the familial (fNPH) and the sporadic groups. Identical questionnaires were sent to the relatives of the potential fNPH patients. Venous blood samples were collected for genetic studies. The disease histories of the probable fNPH patients (n = 60) were compared with their ≥ 60-year-old relatives with no iNPH (n = 49). A modified Charlson Comorbidity Index (CCI) was used to measure the overall disease burden. Fisher’s exact test (two-tailed), the Mann–Whitney U test (two-tailed) and a multivariate binary logistic regression analysis were used to perform the statistical analyses.

**Results:**

Diabetes (32% vs. 14%, p = 0.043), arterial hypertension (65.0% vs. 43%, p = 0.033), cardiac insufficiency (16% vs. 2%, p = 0.020) and depressive symptoms (32% vs. 8%, p = 0.004) were overrepresented among the probable fNPH patients compared to their non-iNPH relatives. In the age-adjusted multivariate logistic regression analysis, diabetes remained independently associated with fNPH (OR = 3.8, 95% CI 1.1–12.9, p = 0.030).

**Conclusions:**

Diabetes is associated with fNPH and a possible risk factor for fNPH. Diabetes could contribute to the pathogenesis of iNPH/fNPH, which motivates to further prospective and gene-environmental studies to decipher the disease modelling of iNPH/fNPH.

## Background

Idiopathic normal pressure hydrocephalus (iNPH) is a chronic and progressive neurological disorder among the elderly [1, 2]. It is characterised by ventriculomegaly in neuroradiological imaging and gait disturbances, while cognitive decline and urinary incontinence are also commonly observed [1, 2]. INPH is treated with a cerebrospinal fluid (CSF) shunt surgery with moderate long-term outcome [3]. In recent meta-analyses, the overall prevalence of iNPH was found to be around 175/100,000 among the elderly and the annual incidence around 1.1–5.5/100,000 [4, 5]. However, a recent prospective population-based study from Sweden found the prevalence of iNPH among the elderly to be as high as 3.7% and increasing with age [6]. Previous studies have suggested cardiovascular risk factors to be associated with the pathology of iNPH [7–15], while the precise pathophysiological basis of iNPH is still unknown [2].

The familial occurrence of iNPH has been previously established [16–26], with some of the drawn pedigrees showing signs of autosomal dominant inheritance. Out of all iNPH patients, 7–16% have been discovered to have symptomatic or shunted relatives [23, 25]. The familial iNPH (fNPH) cases have also been found to slightly differ from the sporadic ones, with potentially more severe symptoms [25]. This all suggests that iNPH could possibly have a heritable form with an independent genetic background or have a familial subgroup, i.e. fNPH [25], but only a few possible risk genes have yet been found. Most promisingly, copy number (CN) loss in intron 2 of the *SFMBT1* gene has been reported as being overrepresented among the iNPH patients in Japanese, Finnish and Norwegian study cohorts [27, 28]. Also, a loss-of-function mutation in *CFAP43* has been found in a Japanese family with multiple iNPH cases [29]. Interestingly, the *SFMBT1* protein has been shown to be present in the structures vital for the CSF dynamics such as the choroid plexus [30], and the *CFAP43*-deficient mice exhibited hydrocephalus and cilia abnormalities [29]. *APOE ε4* is not associated with the development of iNPH but is commonly seen in the iNPH patients with comorbid Alzheimer’s disease (AD) [31, 32].

The aim of this study was to conduct a case-controlled comparison of the fNPH patients to their elderly non-iNPH relatives. By comparing people with similar environmental and genetic backgrounds, important information regarding the comorbidities and the possible risk factors of developing iNPH can be acquired [33]. The possible heritability of iNPH is a notable addition to the previously reported findings regarding the cardiovascular risk factors in iNPH [7–15].

## Methods

### Data collection and the selection of the participants

The collection of the data is described in detail in a previous study that compared the fNPH patients and the sporadic iNPH patients against each other [25]. The same data was used in this study for the fNPH patients with some new cases as the data collection and the patient recruitment were continued afterwards. A retrospective recruitment of the iNPH patients from all neurosurgical units in Finland, shunted since 1993, was performed. The iNPH patients were searched from these registries based on both operative procedure codes and diagnostic codes (ICD 10; G91.2).

Until the June of 2020, altogether 1349 patients were sent a questionnaire inquiring on their medical conditions and possible family symptomatology, from which 718 (53.2%) were returned with informed consent. The medical records of the possible and probable iNPH patients with returned questionnaires were reviewed by the study neurosurgeon of the corresponding unit to exclude secondary normal pressure hydrocephalus (sNPH) [1, 2]. Altogether, 100 patients were discovered to have a potential secondary cause of NPH indicated in the medical records and they were excluded from the study. The final number of the iNPH patients with a returned questionnaire and informed consent was 618 (45.8%) (index patients). The 6-page questionnaire (Additional file 1) contained questions related to iNPH, comorbidities, physical condition, alcohol drinking, smoking and a brief family anamnesis of relatives with a possible iNPH-symptomatology. It also contained questions on medications which were used to cross-check the validity of the answers when available.

INPH was considered potentially familial (fNPH) if the index patient reported at least one relative with ≥ 2 symptoms of the triad or a diagnosed iNPH. These potentially familial iNPH patients were phone-interviewed to exclude the possibility of a known etiology other than iNPH causing the relative’s symptoms. An identical questionnaire was sent to those relatives willing to participate in the study. The relatives were first contacted by the index patient or their next of kin. The questionnaire was sent only to the relatives of those iNPH patients whose iNPH was considered potentially familial. The relatives reporting the triad symptoms were also phone-interviewed about their symptoms.

Out of the 618 iNPH index patients, 96 (15.5%) were found to potentially have a familial iNPH (fNPH), which is in line with previous studies [23, 25]. These potential fNPH patients were found in 79 different families. Altogether 288 relatives were sent the questionnaire and 170 (59.0%) returned it with an informed consent. Approximately three-generation pedigrees were drawn from these fNPH families based on the phone interviews and the questionnaire information. Venous blood samples were also collected from the participants to be used for genetic studies. All participants included in the study were selected independent of the exposure of interest to avoid selection bias [34, 35].

### Categorisation of the patients and their relatives

**Fig. 1:**
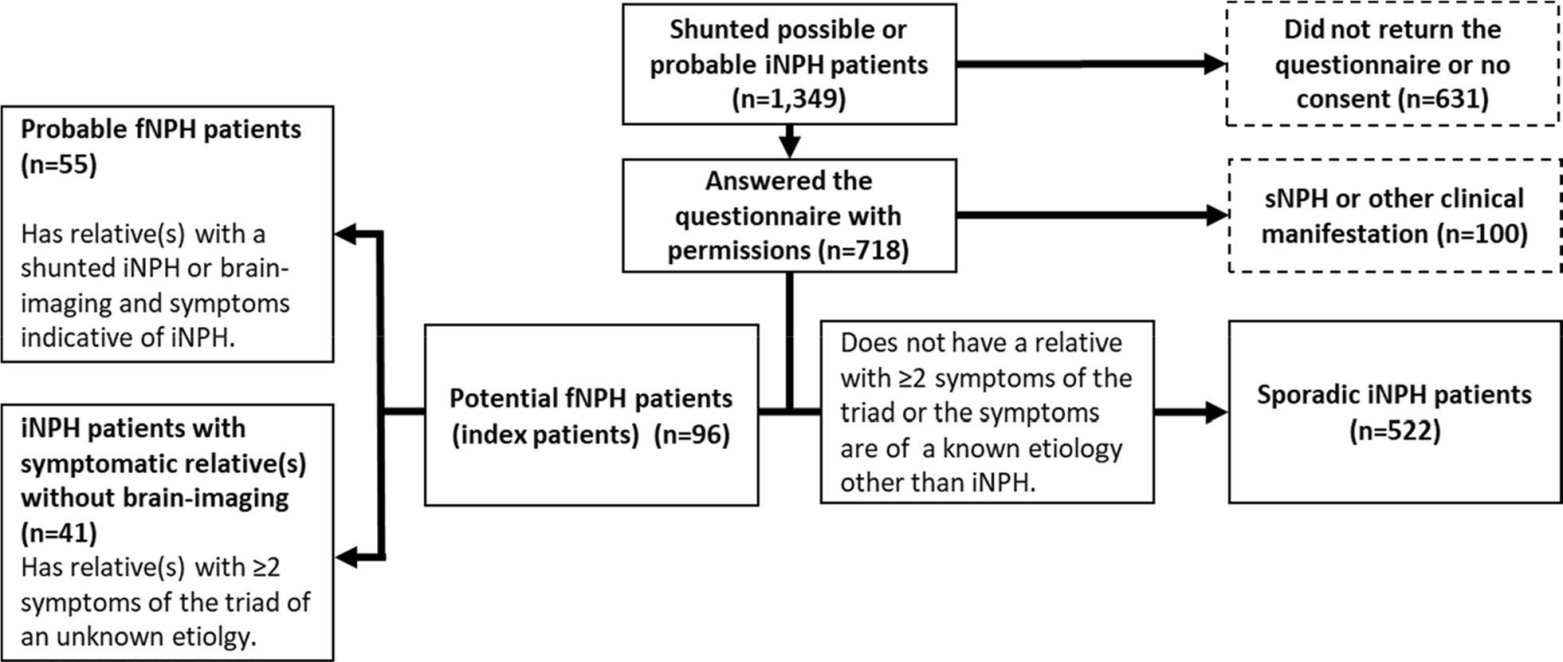
How the shunted possible or probable iNPH patients (index patients) were divided into the familial (fNPH) and the sporadic categories

The potential fNPH index patients were then divided into two categories based on the probability of them truly having multiple iNPH/fNPH cases in the family. A probable fNPH index patient (n = 55) had at least one relative with a diagnosed iNPH or the relative had brain-imaging available with findings (ventriculomegaly, narrowing of the sulci and often disproportionately enlarged subarachnoid space hydrocephalus) and symptoms indicative of iNPH [1, 2]. The number of the probable fNPH families was 39. The remaining patients with potential fNPH were considered iNPH patients with at least one relative with ≥ 2 symptoms of the triad caused by an unknown etiology but the relative had no brain-imaging available to reliably confirm iNPH (n = 41) (Fig. 1). The number of these families was 40.

**Fig. 2:**
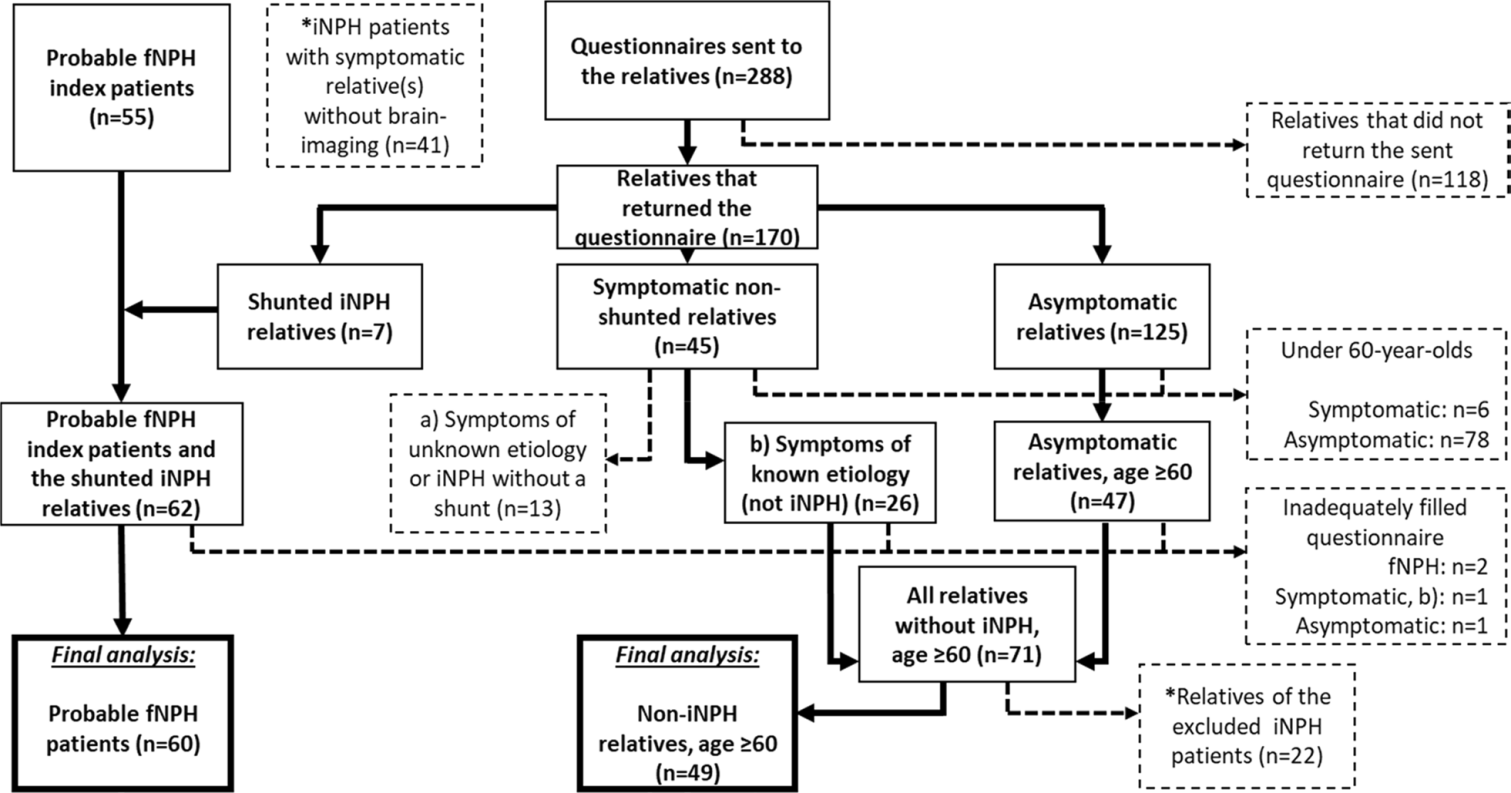
How the compared groups in the final analysis were formed based on the questionnaires and the phone interviews. The dashed lines and the boxes with dashed borders indicate exclusion from the final analysis. The left side of the figure shows how the group of “Probable fNPH patients” (n = 60) was formed from the probable fNPH index patients and their relatives with iNPH and a shunt. Everyone in this group had a diagnosed and shunted iNPH. The middle and the right part of the figure show how the control group of “Non-iNPH relatives, age ≥ 60” (n = 49) was formed from the ≥ 60-year-old relatives of the probable fNPH patients. These relatives were either asymptomatic or had a known etiology for their symptoms other than iNPH. *The iNPH patients with symptomatic relative(s) without brain-imaging and their healthy relatives were excluded from the final analysis

The relatives returning the questionnaire were categorised as symptomatic or asymptomatic, based on whether they had symptoms of the triad, and as the shunted iNPH patients (Fig. 2).

### Formation of comparable groups

The aim of the study was to compare the fNPH patients to their elderly non-iNPH relatives. The groups in the final analysis were “Probable fNPH patients” (n = 60) and the control group of “Non-iNPH relatives, age ≥ 60” (n = 49) (Tables 3 and 4). Only the probable fNPH patients and their relatives were included to the final analysis as these families have multiple iNPH (fNPH) cases that we were able to reliably confirm. The iNPH patients with symptomatic relatives without brain-imaging available to reliably confirm the relative to truly suffer from iNPH were excluded from the final analysis as well as their healthy relatives (Fig. 2).

The group of “Probable fNPH patients” (n = 60) was formed from the probable fNPH index patients (n = 55) and their relatives with a shunted iNPH and a returned questionnaire (n = 7). The medical records of these relatives with shunted iNPH were also re-evaluated to exclude sNPH. Those with inadequately filled questionnaires were excluded (n = 2). All the patients in this category had a diagnosed and shunted iNPH.

The control group of “Non-iNPH relatives, age ≥ 60” (n = 49) consisted of only the relatives of the probable fNPH patients that were ≥ 60 years old, asymptomatic or had a confirmed cause other than iNPH for their symptoms and had an adequately filled questionnaire. The relatives under the age of 60 were excluded to match the age range of the control group with the late average onset age of iNPH. The symptomatic relatives with indefinite triad symptoms or iNPH without a shunt were excluded. Also, those with inadequately filled questionnaires were excluded (Fig. 2). The control group consisted of 19 sisters, 15 brothers, 2 daughters, 1 uncle, 7 nieces and 5 nephews of the probable fNPH index patients.

Summary of the terms related to NPH and the description of different groups are shown in the Table 1 and the iNPH-related characteristics of the probable fNPH patients included in the final analyses are shown in the Table 2.

**Table 1:** Summary of the terms related to normal pressure hydrocephalus (NPH) and the different groups described in the paper

Idiopathic normal pressure hydrocephalus (iNPH)	The idiopathic form of NPH in general (includes both familial and sporadic subgroups)
Familial idiopathic normal pressure hydrocephalus (fNPH)	The familial form of iNPH. An iNPH patient was referred to as a fNPH patient if there were multiple iNPH (fNPH) cases in the family (the precise criteria are described in “Methods” section)
Sporadic idiopathic normal pressure hydrocephalus	An iNPH patient does not have any relatives with iNPH
Secondary normal pressure hydrocephalus (sNPH)	NPH caused by a known (acquired) cause, e.g. subarachnoid hemorrhage, brain tumor, traumatic brain injury etc.
Non-iNPH relatives	In this paper, the probable fNPH patients’ ≥ 60-year-old relatives that had no iNPH (fNPH)

**Table 2:** INPH-related characteristics of the probable fNPH patients (n = 60) included in the final analysis

Self-reported iNPH characteristics	n = 60
Gait disturbances	47/57 (82.5%)
Cognitive decline	39/56 (69.6%)
Urinary incontinence	33/57 (57.9%)
Complete triad	23/60 (38.3%)
Shunt response	52/57 (91.2%)

*fNPH* familial idiopathic normal pressure hydrocephalus

### *APOE* and *SFMBT1* genotyping

Genomic DNA was extracted from the venous blood samples with the QIAamp DNA blood mini extraction kit (QIAGEN). *APOE* was genotyped from 45/60 (75%) of the probable fNPH patients and from 25/49 (51%) of their ≥ 60-year-old non-iNPH relatives by determining 2 single-nucleotide polymorphisms (rs429358 and rs7412) by using the polymerase chain reaction (PCR), the TaqMan genotyping assays (Applied Biosystems (ABI), Foster City, CA, USA) and an allelic discrimination method on the ABI 7000 platform [36]. Possible CN loss in intron 2 of the *SFMBT1* gene was determined from 44/60 (73%) of the probable and fNPH patients and from 22/49 (45%) of their ≥ 60-year-old non-iNPH relatives by using quantitative PCR and the delta–delta method [27, 28].

### Modified Charlson comorbidity index

The overall disease burdens in the compared groups were measured by using the Charlson Comorbidity Index (CCI) [37]. The CCI was slightly modified with some assumptions to better fit our research question and the data that was available from the questionnaires (see Discussion, *Strengths and limitations*). Modifications included: diabetes was considered uncomplicated, cancer was considered unmetastasized, liver disease was considered mild and dementia was excluded.

### Statistical analyses

For the statistical analyses, Fisher’s exact test (two-tailed) was used for the categorical variables and the Mann–Whitney U test (two-tailed) for all continuous variables, as they were abnormally distributed (significance in the Shapiro–Wilk test). The multivariate binary logistic regression analysis was used to account for the confounding between the clinical variables by using the enter method. The clinical variable was included to the age adjusted multivariate model if (1) it had a *p* value of < 0.05 in the Fisher’s exact test, (2) it was considered fNPH comorbidity, (3) the comorbidity could potentially affect the pathogenesis of fNPH. Correlation was tested by using the Pearson correlation coefficient. The variables in the analyses were based on the data in the questionnaires and the phone interviews, apart from the genomic data. The categorical variables were mainly dichotomous. P < 0.05 was considered statistically significant. SPSS statistical software (version 22.0, SPSS INC, Chicago, Illinois) was used to perform the statistical analyses.

## Results

The mean age of the probable fNPH patients was higher compared to their non-iNPH relatives (76.9 vs. 70.0, p < 0.001). Arterial hypertension (65% vs. 43%, p = 0.033), diabetes (32% vs. 14%, p = 0.043), cardiac insufficiency (16% vs. 2%, p = 0.020) and depressive symptoms (32% vs. 8%, p = 0.004) were overrepresented among the probable fNPH patients compared to their non-iNPH relatives. The probable fNPH patients were less likely to consume alcohol than their non-iNPH relatives (32% vs. 63%, p = 0.001). Only 46% of the probable fNPH patients filled the questionnaire independently compared to 88% of the non-iNPH relatives (Table 3). The age-adjusted multivariate logistic regression analysis included diabetes, cardiac insufficiency and arterial hypertension as these comorbidities were considered to potentially contribute to the pathogenesis of iNPH. Only a weak correlation was found between these four clinical variables (Pearson correlation coefficient, R < 0.34). In the multivariate analysis, age at questionnaire (OR = 1.1, 95% CI 1.1–1.2, p < 0.001) and diabetes (OR = 3.8, 95% CI 1.1–12.9, p = 0.030) remained independently significant (Table 4).

**Table 3:** Comparison of questionnaire data between the probable fNPH patients (n = 60) vs. their ≥ 60-year-old non-iNPH relatives (n = 49), from a total of 39 different families

	Probable fNPH patients (n = 60)	Non-iNPH relatives, age ≥ 60 (n = 49)	p-value
Mean age at questionnaire (± SD)	76.9 (± 7.4)	70.0 (± 8.4)	*<* *0.001*^**a**^
Sex (F/M)	32/28 (53.3%)	28/21 (57.1%)	0.704^b^
Mean BMI (± SD)	27.8 (± 4.4)	27.5 (± 5.3)	0.501^a^
Smoking and alcohol			
Smoker or ex-smoker	16/60 (26.7%)	18/49 (36.7%)	0.302^b^
Consumes alcohol	19/60 (31.7%)	31/49 (63.3%)	*0.001*^*b*^
Prevalence of			
*APOE* ε4	10/45 (22.2%)	8/25 (32.0%)	0.403^b^
Loss of CN in intron 2 of *SFMBT1*	4/44 (9.1%)	2/22 (9.1%)	1.000^b^
Memory and neurological comorbidities			
Diagnosed AD	10/60 (16.9%)	2/49 (4.1%)	0.061^b^
Parkinsonism	2/60 (3.3%)	0/49 (0.0%)	0.501^b^
Other diagnosed neurodegenerative disorder	2/59(3.4%)	1/49 (2.0%)	1.000^b^
Epilepsy	5/60 (8.3%)	1/49 (2.0%)	0.220^b^
Cardiovascular comorbidities			
Arterial hypertension	39/60 (65.0%)	21/49 (42.9%)	*0.033*^*b*^
Diabetes	19/60 (31.7%)	7/49 (14.3%)	*0.043*^*b*^
Coronary artery disease	8/58 (13.8%)	2/48 (4.2%)	0.108^b^
Myocardial infarction	2/58 (3.4%)	0/49 (0.0%)	0.499^b^
Cardiac insufficiency	9/58 (15.5%)	1/49 (2.0%)	*0.020*^*b*^
Cardiac arrhythmia	13/58 (22.4%)	8/41 (16.3%)	0.473^b^
Venous thrombosis	6/59 (10.2%)	2/48 (4.2%)	0.292^b^
Stroke/TIA	2/58 (3.4%)	2/49 (4.1%)	1.000^b^
Other comorbidities			
Rheumatoid arthritis	2/59 (3.4%)	2/47 (4.3%)	1.000^b^
Spinal stenosis	11/58 (19.0%)	3/48 (6.3%)	0.082^b^
Depressive symptoms	19/59 (32.2%)	4/49 (8.2%)	*0.004*^*b*^
Other mental disease	4/59 (6.8%)	1/49 (2.0%)	0.374^b^
Asthma	11/60 (18.3%)	9/49 (18.4%)	1.000^b^
COPD	3/60 (5.0%)	1/49 (2.0%)	0.626^b^
Peptic ulcer	4/60 (6.7%)	1/49 (2.0%)	0.376^b^
Hypothyroidism	7/58 (12.1%)	6/49 (12.2%)	1.000^b^
Chronic snoring	12/60 (20.0%)	10/49 (20.4%)	1.000^b^
Sleep apnea	3/59 (5.1%)	3/48 (6.3%)	1.000^b^
Mean modified CC score (± SD)	0.95 (± 1.06)	0.75 (± 1.12)	0.336^a^
Performance			
Is able to fill the questionnaire independently	27/59 (45.8%)	43/49 (87.8%)	*<* *0.001*^*b*^

Italic values indicate significance of *p* value (< 0.05)

The questionnaire included more data but only the more interesting findings are shown in this table

*fNPH* familial idiopathic normal pressure hydrocephalus, *SD* standard deviation, *F/M* female/male, *BMI* body mass index, *AD* Alzheimer’s disease, *TIA* transient ischemic attack, *COPD* chronic obstructive pulmonary disease, *CCI* Charlson comorbidity index

^a^Mann-Whitney U test (two-tailed)

^b^Fisher’s exact test (two-tailed)

**Table 4:** Logistic regression analysis comparing the probable fNPH patients (n = 60) and their non-iNPH relatives, age ≥ 60 (n = 49) as a reference category

Clinical variable	n	Model	OR	95% CI	p-value
Age at questionnaire	109	Univariate	1.121	1.057–1.189	*<* *0.001*
	107	Multivariate	1.123	1.061–1.189	*<* *0.001*
Diabetes	109	Univariate	2.780	1.057–7.317	*0.038*
	107	Multivariate	3.840	1.142–12.912	*0.030*
Cardiac insufficiency	107	Univariate	8.816	1.075–72.282	*0.043*
	107	Multivariate	4.250	0.475–38.030	0.196
Arterial hypertension	109	Univariate	2.476	1.140–5.378	*0.022*
	107	Multivariate	1.147	0.444–2.959	0.777

Italic values indicate significance of *p* value (< 0.05)

Hosmer–Lemeshow = 0.189; Overall percentage = 75.7%

*fNPH* familial idiopathic normal pressure hydrocephalus, *OR* odds ratio, *CI* confidence interval

No significant differences were found in the prevalence of *APOE* ε4 (22% vs 32%, p = 0.403) or the CN loss in intron 2 of *SFMBT1* (9% vs 9%, p = 1.000) (Table 3), but diabetes was present in 3 out of the 4 probable fNPH patients that had CN loss in the *SFMBT1* gene compared to none out of 2 of the non-iNPH relatives.

## Discussion

### Diabetes

The most important finding of this study is the tendency towards increased prevalence of the cardiovascular comorbidities, especially diabetes, in the fNPH patients compared to their non-iNPH relatives. Although the age difference between the two groups was nearly 7 years, diabetes remained independently significant in the multivariate model when adjusted to age, whereas arterial hypertension or cardiac insufficiency did not. Previous studies have compared the differences in the prevalence of diabetes between the iNPH patients and the healthy controls with comparable age distribution [7–14]. Using a table from the review by Hudson et al. [38] that summarized the results of seven of these studies, we can calculate the pooled prevalence of diabetes among the iNPH patients and the controls. In these seven studies the pooled diabetes rates in iNPH were 24% compared to 10% in the controls, prevalence ratio 2.4:1, p < 0.001 (χ2-test) (only 70–90-year-olds included from the Eide and Pripp’s study [11]). Our results with the novel study design closely agree with these previous results when it comes to iNPH (32% vs. 14%; prevalence ratio 2.3:1). Additionally, in our previous study [25], no significant differences were found in the prevalence of diabetes between the sporadic iNPH and the fNPH patients.

Other cardiovascular risk factors, including arterial hypertension, dyslipidemia, obesity and physical inactivity have been also found to be overrepresented in the iNPH patients [7, 9–13, 15], suggesting that they could be possible risk factors for the development of iNPH. This is also backed by the finding of cerebral microbleeds being detected more often in the iNPH patients in magnetic resonance imaging (MRI), and thus a vascular component could possibly affect the pathophysiology of iNPH [39]. A recent study that compared four different types of adult hydrocephalus (transitional, unrecognized congenital, acquired and iNPH) found out that the prevalence of cardiovascular comorbidities in iNPH was significantly higher compared to the other types [40]. This finding together with the later onset age of iNPH indicates that the cardiovascular comorbidities could have a chronic effect on its development.

There is evidence that the glymphatic system dysfunction could affect the development of iNPH [41–43]. It has been suggested that in iNPH the glymphatic system is possibly impaired through neuroinflammation, reactive astrogliosis, depolarization and reduced density of aquaporin-4 (AQP4) and sleep disturbances, which could reduce the normal clearance of CSF [43–45]. Interestingly in rat models, diabetes has been found to cause glymphatic system dysfunction, reduction in AQP4 density, neuroinflammation, microvascular damage, blood–brain barrier damage and cognitive decline that could be associated with glymphatic system dysfunction [46–49]. It seems that diabetes could also cause astrogliosis and dysregulated metabolism in astrocytes in mouse and rat models [49]. By affecting the astrocytes diabetes has also been found to reduce the glutamate uptake in brain in rat models [50, 51]. Interestingly, iNPH patients have been found to suffer from corticospinal hyperexcitability and it has been hypothesized to possibly derive from increased activity of glutamatergic system [52, 53].

Some studies have also found the iNPH patients to suffer from a decreased cerebral metabolic rate of glucose [54], reduced thalamic N-acetylaspartate and total N-acetylaspartate, an important metabolite in the central nervous system [55], and the down-regulation of the adenosine receptors that together with adenosine are important for the vascular protection and the modulation of inflammation [56]. This together with the high prevalence of cardiovascular comorbidities shows that metabolic dysfunction seems to be present in iNPH and potentially also in fNPH. On the other hand, it has also been suggested that diabetes in iNPH could be a consequence of ventriculomegaly and compression damage to the hypothalamic pituitary axis causing hormonal imbalances [38].

The questionnaire did not classify the type of diabetes the participants had. We can assume that nearly all of the cases were type 2 diabetes (T2DM) since the overall prevalence of T2DM among the elderly is remarkably higher than type 1 diabetes (T1DM) [57]. We would expect the rationale about iNPH/fNPH, cardiovascular risk factors and diabetes to hold true at least in T2DM, T1DM and latent autoimmune diabetes in adults (LADA) but there seems to be only very few studies concerning NPH and the different types of diabetes other than T2DM. One reason could be that the life expectancy of a patient with T1DM used to be quite poor in the past compared to the average onset age of iNPH [58]. In one study, a possible presence of NPH was found in 6 insulin-dependent diabetic patients with recurrent hypoglycemic coma (mean age 62, mean diabetes duration 25 years) [59]. Their diabetes types were not precisely classified in the study but most likely either T1DM, LADA or progressed T2DM.

These findings support the idea that diabetes could impact the development of iNPH and fNPH and even its phenotype. However, it is unclear how significant this impact is as the majority of iNPH patients do not seem to have diabetes although it being clearly overrepresented in iNPH compared to the general population. It is also unclear whether the treatment or the prevention of certain metabolic dysfunctions or the cardiovascular comorbidities would effectively prevent the development of iNPH/fNPH or if there were other factors affecting it. Especially the potential genetic aspect of diabetes in iNPH/fNPH is intriguing and warrants further research.

### Depressive symptoms

Symptoms of depression have been found to be common among the iNPH patients in previous studies [60–62], which is in line with our findings. Depression itself is probably not an independent risk factor for the development of iNPH but more likely a result of the increased disability due to iNPH or other comorbidities that may contribute to the pathogenesis of iNPH or to the development of depression itself [60–62]. It might be that iNPH is associated with an extensive range of psychiatric symptoms [14, 15] supported by our recent report indicating that schizophrenia is more common in the iNPH patients compared to the general population [63]. Further prospective studies regarding the symptoms of depression and iNPH are needed.

### The identified relatives

When it comes to the excluded iNPH patients with symptomatic relatives that had no brain-imaging available to confirm the relative’s iNPH, the family history is usually based on either symptomatic mother, father or sibling that has already died. It is plausible that some of these potential fNPH cases are actually sporadic. After all, it would be interesting to study both symptomatic and asymptomatic relatives of iNPH patients regardless of the prior family history on potential NPH-related symptoms, although the probability of finding genetic risk factors could be notably higher in those with clear family history. A consensus on determining the diagnosis of fNPH is needed considering that full consensus of definite iNPH diagnosis is actually also lacking.

The pedigrees offer a novel opportunity to study the genetics and the pathophysiology of iNPH/fNPH. In addition to this, when we learn more about the development of iNPH, it allows us to possibly detect the relatives who are at a greater risk of developing iNPH and to potentially achieve a preclinical diagnosis of iNPH, as iNPH seems to show signs of asymptomatic ventriculomegaly (AVIM) in the neuroradiological imaging years before the clinical symptoms appear [64, 65]. This could be important since delayed shunting seems to hamper the clinical outcome of iNPH [66]. INPH is quite an unknown disorder among the general population but the knowledge of a possible familial aggregation of iNPH (fNPH) might allow the relatives to detect the symptoms of NPH more easily and to potentially seek treatment before the symptoms progress severely.

### Alcohol, sleep apnea, *SFMBT1* and *APOE ε4*

Alcohol consumption was recently suggested to be a potential risk factor for iNPH in two studies [14, 67]. Our results don’t back up this finding, but it must be noted that our questionnaire represents only the time close to the diagnosis and not their alcohol consumption earlier in life. Also, a frequent association between iNPH and obstructive sleep apnea has been found [45]. Our analysis with the probable fNPH families showed no differences in the prevalence of sleep apnea between the groups (Table 3).

An interesting finding in the study was the similar prevalence of the CN loss in intron 2 of the *SFMBT1* gene between the fNPH patients and their non-iNPH relatives (9% vs. 9%), despite the allelic variation in *SFMBT1* being discovered to be overrepresented in the iNPH patients in a Japanese study cohort [27] and also in Finnish and Norwegian cohorts [28]. This is the first time the *SFMBT1* CN loss has been directly compared between the fNPH patients and their relatives. Korhonen et al. [28] found the *SFMBT1* CN loss to be present in 5% of the general Finnish population, which is less than it was in the non-iNPH relatives of these probable fNPH patients. We can speculate whether the *SFMBT1* CN loss accumulates in these families exposing them to a greater risk of developing iNPH. The *SFMBT1* CN loss might require some other unknown external factor to trigger the development of iNPH, and interestingly in this study, diabetes was present in 3 out of the 4 probable fNPH patients that had CN loss in the *SFMBT1* gene compared to none out of 2 of the non-iNPH relatives. This indicates that diabetes might be one potential trigger that is needed for the CN loss in intron 2 of *SFMBT1* to cause iNPH and raises a question for further study on the potential gene-environmental interactions. The brain MRIs of these elderly relatives with the CN loss in *SFMBT1* in this study would be beneficial to exclude the possibility of asymptomatic ventriculomegaly [64, 65]. It must be noted that the number of the *SFMBT1* genotyped relatives in this study is small, so pure coincidence could have possibly affected the results. More studies concerning the mechanism between *SFMBT1* and iNPH are needed.

*APOE* ε4 did not show any association with fNPH when compared to the relatives (22% vs. 32%). This is in-line with the previous findings [31, 32] and strengthens the assumption that fNPH has a genetic background independent from AD. From the probable fNPH patients suffering from comorbid AD, 50% were carriers of *APOE* ε4 in this study group.

### Strengths and limitations

The main strengths of this study are that only the probable fNPH families with multiple confirmed cases were included in the analysis and the families came from a fairly homogeneous population. The questionnaires were well-filled as the modified CCI scores were measurable from 93% of the probable fNPH patients and 98% of the non-iNPH relatives included in the final analysis. The questionnaires sent to the patients and to their relatives were also identical and therefore the results were closely comparable.

There are limitations and potential sources of error. The data used was based on the questionnaires that were filled by the participants themselves or by their next of kin, which might create a potential source of error. The phone-interview-based data recruitment of the relatives to the study is not the most effective and reliable method. Due to the nature of iNPH, dementia was excluded from the modified CCI as it would probably have caused bias. The variables in the study were mainly dichotomous, and therefore assumptions concerning diabetes and cancer in the CCI measurements were made as we have little information about the severity of the comorbidities, which requires further study.

### Significance of the research and future perspectives

This is the first comparison of familial iNPH patients and their elderly non-iNPH relatives that includes multiple families. This data is important when deciphering the pathophysiology and the genetics behind iNPH in the future. These results show that the fNPH patients and their elderly non-iNPH relatives seem to differ from each other, and therefore there must be an as-yet-unknown explanation for why some of the family members develop iNPH while the others do not. This study also supports the previous findings that the overrepresentation of cardiovascular comorbidities and depressive symptoms are associated with iNPH, as being conducted from a familial standpoint. Due to our small sample size, it would be beneficial to replicate these findings with a larger sample size. Considering the relative rarity of fNPH international multicentre studies are needed in the future.

## Conclusions

Diabetes was independently associated with fNPH in the study. The diabetes rates were in-line with the previous iNPH studies. Diabetes has previously been found to cause neuroinflammation, altered brain metabolism and microvascular damage. It has also been found to impair the glymphatic system and to cause reduction in AQP4 density in rat models, which could disturb the normal clearance of the CSF. Therefore, diabetes might be an independent risk factor for the development of iNPH/fNPH. It is unclear how effective the treatment or the prevention of diabetes itself would be in preventing the possible development of iNPH or improving the outcome of a shunt surgery. It is also unclear what is the impact of potential gene-environmental interactions when it comes to diabetes and iNPH/fNPH. The identified pedigrees offer a novel opportunity to study the genetics behind iNPH and to possibly achieve preclinical diagnoses of iNPH.

## Supplementary information

**Additional file 1.** NPH questionnaire. The questionnaire that was used in the study. All the questionnaires that were sent to the participants were in Finnish.

## Data Availability

The datasets generated and/or analysed during the current study are not publicly available as they include extensive personal data but are available from the corresponding author upon reasonable request.

## Abbreviations

iNPH: Idiopathic normal pressure hydrocephalus
fNPH: Familial idiopathic normal pressure hydrocephalus
CSF: Cerebrospinal fluid
CN: Copy number
AD: Alzheimer’s disease
sNPH: Secondary normal pressure hydrocephalus
PCR: Polymerase chain reaction
CCI: Charlson Comorbidity Index
MRI: Magnetic resonance imaging
T2DM: Type 2 diabetes
T1DM: Type 1 diabetes
LADA: Latent autoimmune diabetes in adults
AVIM: Asymptomatic ventriculomegaly
AQP4: Aquaporin-4
