# Supplementary material for: Diabetes is associated with familial idiopathic normal pressure hydrocephalus: a case–control comparison with family members
Source: Fluids Barriers CNS. 2020 Sep 15;17:57. doi: 10.1186/s12987-020-00217-0 (PMC7493374; doi:10.1186/s12987-020-00217-0)
Supplement: Supplementary file 1 — Additional file 1. NPH questionnaire. The questionnaire that was used in the study. All the questionnaires that were sent to the participants were in Finnish. [file 12987_2020_217_MOESM1_ESM.docx]

| **We politely ask You or your next of kin to carefully fill this questionnaire at home and return it in the envelope (the postal charge has already been paid).**  **The questions are answered by myself / next of kin**  If by the next of kin, his/her name, relationship and phone number  ______________________________________________________________ | | | | | | | | | | | | | | | | | | | | | | | | | |
| --- | --- | --- | --- | --- | --- | --- | --- | --- | --- | --- | --- | --- | --- | --- | --- | --- | --- | --- | --- | --- | --- | --- | --- | --- | --- |
| **GENERAL INFROMATION** | | | | | | | | | | | | | | | | | | | | | | | | | |
|  | | | | | | | | | | | | | | | | | | | | | | | | | |
| **PERSONAL DATA** | | | | | | | | | | | | |  | | | | | | | | | | | | |
| Name | | | | | | | | | | | | | Hometown | | | | | | | | | | | | |
|  | | | | | | | | | | | | |  | | | | | | | | | | | | |
| Social security number | | | | | | | | | | | | | Address | | | | | | | | | | | | |
| Place of birth | | | | | | | | | | | | | Phone number | | | | | | | | | | | | |
|  | | | | | | | | | | | | |  | | | | | | | | | | | | |
| **HEALTH INFROMATION** | | | | | | | | | | | | |  | | |  | | | | | | | | | |
|  | | | | | | | | | | | | |  | | |  | | | | | | | | | |
| Height | |  |  | | Weight |  | | | | |  | | | | |  | | | | | |  | |  | |
|  | |  |  | |  |  | | |  | | | | | | |  | | | | | |  | |  | |
| Smoking? | | | No | | | | | | | | | | | | | Alcohol use? | | | | | | No | | Yes | |
|  | | | Not anymore, quit year | | | | | | | |  | |  | | | amount | | |  | portions/week | | | |  | |
|  | | | Yes, amount | | | | |  | | cigarettes/per day | | | | | | 1 portion= 1 bottle of beer, a glass of wine or 4 cl of strong alcohol | | | | | | | | | |
|  | | |  | | | | |  | |  | | | | | |  | | | | | | |  |  | |
| **PHYSICAL PERFORMANCE** | | | | | | | | | | | | | | | | | | | | | | | | | |
|  | | | | | | | | | | | | | | | | | | | | | | | | | |
| How many floors can you ascend without stopping? | | | | | | | | | | | | | | | | | | | | | | | | | |
|  | | | |  | | |  | | | |  | | | |  | | | | | | | | | | |
| None | | | | 1 | | | 2 | | | | 3 | | | | over 3 | | | | | | | | | | |
|  | | | | | | | | | | | | | | |  | |  | | | | | | | | |
| Can you walk uphill without stopping? | | | | | | | | | | | | | | | Yes | | No | | | | | | | | |
|  | | | | | | | | | | | | | | |  | |  | | | | | | | | |
| What sort of physical activity can you do regularly? | | | | | | | | | | | | | | | | | | | | | | | | | |
|  |  | | | | | | | | | | | | | | | | | | | | | | | |  |
|  |  | | | | | | | | | |  | | | |  | | | | | | | | | |  |
|  | | | | | | | | | | |  | | | |  | | | | | | | | | | |
|  | | | | | | | | | | |  | | | |  | | | | | | | | | | |
| Limitations to my movement | | | | | | Chest pain | | | | | | | | | No | | | Yes | | | | | | | |
|  | | | | | | Lower limb pain | | | | | | | | | No | | | Yes | | | | | | | |
|  | | | | | | Shortness of breath | | | | | | | | | No | | | Yes | | | | | | | |
|  | | | | | | Other, what? | | | | | |  | | |  | | |  | | | | | | | |
| I can wash myself and get dressed independently | | | | | | | | | | | | | | | | | No | | | | Yes | | | | |
| I can do chores at home independently | | | | | | | | | | | | | | | | | No | | | | Yes | | | | |
| I can take care of my affairs outside the home | | | | | | | | | | | | | | | | | No | | | | Yes | | | | |
| I can fill this questionnaire independently | | | | | | | | | | | | | | | | | No | | | | Yes | | | | |
|  | | | | | | | | | | | | | | | | |  | | | |  | | | | |
| I live | | alone | | | with partner/family | | | | | | | | | in nursing home | | | | | | | other, how? | | | | |
|  | |  | | |  | | | | | | | | | |  | | | | | |  | | | | |
|  | |  | | |  | | | | | | | | | |  | | | | | |  | | | | |

| **DISEASES** | | | | | | | | | | | |
| --- | --- | --- | --- | --- | --- | --- | --- | --- | --- | --- | --- |
| Do you have / have you had any of the following diseases or conditions? | | | | | | | | | | | |
|  | | | | | | | | | | | |
| Arterial hypertension | | | No | Yes | Asthma | | | No | | Yes | |
| Coronary artery disease | | | No | Yes | COPD | | | No | | Yes | |
| Myocardial infarction, year | |  | No | Yes | Other lung disease, what? | | | No | | Yes | |
| Cardiac insufficiency | | | No | Yes |  | | |  | |  | |
| Cardiac arrhythmia | | | No | Yes | Chronic snoring | | | No | | Yes | |
| Heart valve disease | | | No | Yes | Sleep apnea | | | No | | Yes | |
| Heart valve prosthesis | | | No | Yes | Alzheimer’s disease | | | No | | Yes | |
| Pacemaker | | | No | Yes | Epilepsy | | | No | | Yes | |
| Other heart disease, what? | | | No | Yes | Parkinson’s disease | | | No | | Yes | |
|  | | |  |  | MS-disease | | | No | | Yes | |
| Peripheral artery blockage | | | No | Yes | Muscular disease | | | No | | Yes | |
| Stroke/TIA | | | No | Yes | Dementia | | | No | | Yes | |
| Vascular prosthesis/stent | | | No | Yes | Developmental disability | | | No | | Yes | |
| where? | | |  |  | Other neurological disease, | | | No | | Yes | |
|  | | |  |  | what? | | |  | |  | |
| Venous cathether | | | No | Yes |  | | |  | |  | |
| Venous thrombosis | | | No | Yes | Deep brain stimulator | | | No | | Yes | |
|  | | |  |  | Cerebrospinal shunt | | | No | | Yes | |
| Pulmonary embolism | | | No | Yes |  | | |  | |  | |
| Varices | | | No | Yes | Cochlear implant | | | No | | Yes | |
| Hemorrhagic tendency | | | No | Yes | Peptic ulcer | | | No | | Yes | |
| Anemia | | | No | Yes | Reflux disease | | | No | | Yes | |
| Other coagulation disorder, what? | | | No | Yes | Other gastrointestinal disease, what? | | | No | | Yes | |
|  | | |  |  |  | | |  | |  | |
|  | | |  |  |  | | |  | |  | |
| Other blood disease, what? | | | No | Yes | Tuberculosis | | | No | | Yes | |
|  | | |  |  | Bacteria MRSA / VRE / ESBL | | | No | | Yes | |
| Liver cirrhosis | | | No | Yes | Hepatitis A / B / C, HIV | | | No | | Yes | |
| Other liver disease,  what? | | | No | Yes | Other contagious disease, what? | | | No | | Yes | |
|  | | |  |  |  | | |  | |  | |
| Renal insufficiency | | | No | Yes | Hypothyroidism | | | No | | Yes | |
| Rheumatoid arthritis | | | No | Yes |  | | |  | |  | |
| Other rheumatic disease, what? | | | No | Yes | Diabetes | | | No | | Yes | |
|  | | |  |  | Cancer, what? | | | No | | Yes | |
| Severe arthrosis | | | No | Yes |  | | |  | |  | |
| Back pain radiating to leg | | | No | Yes | Organ transplant, what? | | | No | | Yes | |
|  | | |  |  |  | | |  | |  | |
| Spinal stenosis | | | No | Yes | Depressive symptoms | | | No | | Yes | |
| Other skeletal disease | | | No | Yes | Other mental disease | | | No | | Yes | |
| what |  | |  |  | what? | |  |  | |  | |
| Limb prosthesis | | | No | Yes | Other diagnosed or suspected disease, | | | No | | Yes | |
| Chronic pain problem | | | No | Yes | what? |  | |  | |  | |
|  | | |  |  |  |  | |  | |  | |
| Pain stimulator | | | No | Yes | I feel myself healthy | | | No | | Yes | |
|  | | |  | | | | |  | |  | |
| Has your condition changed during the past three months? | | | | | | | | No | | Yes | |
| If yes, how? | | | | | | | | | | | |
|  | | | | | | | | | | | |
|  | | | | | | | | | | | |
|  | | | | | | | | |  | |  |
|  | | | | | | | | |  | |  |

| **SURGERIES, GENERAL AND REGIONAL ANESTHESIA** | | | | | | |
| --- | --- | --- | --- | --- | --- | --- |
| What surgery/operation; general or regional anesthesia, year? | | | | | | |
|  | | | | | | |
|  | | | | | | |
|  | | | | | | |
|  | | | | | | |
|  | | | | | | |
|  | | | | | | |
| **MEDICATION** | | |  |  | |  |
| Blood pressure medication (name of the medicine, dosage): | | | | | | |
|  | | | | | | |
|  | | | | | | |
|  | | | | | | |
| Diabetes medication (name of the medicine, dosage): | | | | | | |
|  | | | | | | |
|  | | | | | | |
|  | | | | | | |
| Pain medication (name of the medicine, dosage):  Alzheimer medication (name of the medicine, dosage):  Other medication (name of the medicine, dosage): | | | | | | |
|  | | | | | | |
| Other medication you use occasionally? | | | | | | |
|  | | | | | | |
| Do you use any natural products? | | | | | | |
|  | | | | | | |
|  | | | | | | |
|  | | | | | | |
| Do you use antithrombotic or anticoagulant medication? | | | | |  | |
|  | Primaspan, Disperin, Asperin, Asasantin | | | |  | |
|  | Plavix, Klopidogrel, Pradaxa, Xarelto | | | | Marevan | |
|  |  | | | |  | |
| Other, what? | |  | | | | |
|  | |  | | | | |
| Why has the medication been prescribed to you? | | | | | | |
|  | | | | | | |
|  | | | | | | |

**NORMAL PRESSURE HYDROCEPHALUS (NPH) RELATED QUESTIONS**

**Circle your answer**

1. I have a shunt (permanent tube from the brain ventricle to peritoneum or pericardium) No Yes

What year was the shunt placed? _______

2. I feel that my condition improved after the shunt was placed. No Yes

3. I have urinary incontinence No Yes

If yes, I need diapers daily / occasionally / not at all (circle most suitable)

If yes, when did the symptoms begin: ___ _______(month/year)

4. I have gait or walking problems No Yes

If yes, when did the symptoms begin: ___ _______(month/year) and

circle the alternatives that best describe your walking: Shuffling

Swaying

Wide-based

5. I use some sort of mobility aid No Yes

If yes, what: ___________________________________________________

6. I need other person’s help to move No Yes

**Memory**

7. I have memory or cognitive problems No Yes

If yes, when did the symptoms begin: ___ _______(month/year)

8. The memory problems cause me harm No Yes

9. I have been diagnosed with Alzheimer’s disease

If yes, what year ____ No Yes

10. I have been diagnosed with some other memory disease? No Yes

If yes, what? ____________________________year_____

11. I have a memory medication for my memory disease No Yes

If yes, what medication? __________________________________________

__________________________________________

**Need for assistance**

12. I need assistance to do chores at home No Yes

13. I need assistance to take care of my affairs outside home No Yes

**FAMILY**

14. **One or more of my relatives have been diagnosed with NPH** **No Yes**

**If yes, fill the information about the relatives that have been diagnosed with NPH carefully (IMPORTANT).**

Does the relative Relative’s contact

1. Name of the relative Relationship Year of birth give us permission information and - (and year of death) to contact him/her phone number

|  |  |  | YES / NO |  |
| --- | --- | --- | --- | --- |

Does the relative Relative’s contact

1. Name of the relative Relationship Year of birth give us permission information and - (and year of death) to contact him/her phone number

|  |  |  | YES / NO |  |
| --- | --- | --- | --- | --- |

Does the relative Relative’s contact

1. Name of the relative Relationship Year of birth give us permission information and - (and year of death) to contact him/her phone number

|  |  |  | YES / NO |  |
| --- | --- | --- | --- | --- |

15. **One or more of my relatives have the same kind of NPH-related symptoms as I do** (e.g. gait problems or memory problems).

**No Yes**

If **yes**, fill the information about the relatives carefully.

Does the relative Relative’s contact

1. Name of the relative Relationship Year of birth give us permission information and - (and year of death) to contact him/her phone number

|  |  |  | KYLLÄ / EI |  |
| --- | --- | --- | --- | --- |

| \|  \| \| --- \| \|  \| \|  \|   Urinary incontinence/ increased urinary frequency  Gait problems  Memory problems |
| --- | --- | --- | --- |

Choose, what symptoms the relative has:

Does the relative Relative’s contact

1. Name of the relative Relationship Year of birth give us permission information and - (and year of death) to contact him/her phone number

|  |  |  | YES / NO |  |
| --- | --- | --- | --- | --- |

| \|  \| \| --- \| \|  \| \|  \|   Urinary incontinence/ increased urinary frequency  Gait problems  Memory problems |
| --- | --- | --- | --- |

Choose, what symptoms the relative has:

Does the relative Relative’s contact

1. Name of the relative Relationship Year of birth give us permission information and - (and year of death) to contact him/her phone number

|  |  |  | YES / NO |  |
| --- | --- | --- | --- | --- |

| \|  \| \| --- \| \|  \| \|  \|   Urinary incontinence/ increased urinary frequency  Gait problems  Memory problems |
| --- | --- | --- | --- |

Choose, what symptoms the relative has:

Continue if necessary.Free word: __________________________________________________

__________________________________________________________________

__________________________________________________________________

__________________________________________________________________

__________________________________________________________________

__________________________________________________________________

__________________________________________________________________

__________________________________________________________________

__________________________________________________________________

__________________________________________________________________

__________________________________________________________________

Please check that you have answered all the questions. Thank you for your effort!
